# Mid-Neolithic Exploitation of Mollusks in the Guanzhong Basin of Northwestern China: Preliminary Results

**DOI:** 10.1371/journal.pone.0058999

**Published:** 2013-03-18

**Authors:** Fengjiang Li, Naiqin Wu, Houyuan Lu, Jianping Zhang, Weilin Wang, Mingzhi Ma, Xiaohu Zhang, Xiaoyan Yang

**Affiliations:** 1 Key Laboratory of Cenozoic Geology and Environment, Institute of Geology and Geophysics, Chinese Academy of Sciences, Beijing, China; 2 Shaanxi Provincial Institute of Archaeology, Xi'an, China; 3 Institute of Cultural Relics in Henan Province, Zhengzhou, China; 4 Institute of Geographic Sciences and Natural Resources Research, Chinese Academy of Sciences, Beijing, China; University of Florence, Italy

## Abstract

Mollusk remains are abundant in archaeological sites in the Guanzhong Basin of Northwestern China, providing good opportunities for investigations into the use of mollusks by prehistoric humans. Here we report on freshwater gastropod and bivalve mollusks covering the time interval from about 5600 to 4500 cal. yrs BP from sites of Mid-Late Neolithic age. They are identified as *Cipangopaludina chinensis* and *Unio douglasiae*, both of which are currently food for humans. The shells are well preserved and have no signs of abrasion. They are all freshwater gastropods and bivalves found in pits without water-reworked deposits and have modern representatives which can be observed in rivers, reservoirs, and paddy fields in the studied region. Mollusk shells were frequently recovered in association with mammal bones, lithic artifacts, and pottery. These lines of evidence indicate that the mollusks are the remains of prehistoric meals. The mollusk shells were likely discarded into the pits by prehistoric humans after the flesh was eaten. However, these mollusk remains may not have been staple food since they are not found in large quantities. Mollusk shell tools and ornaments are also observed. Shell tools include shell knives, shell reaphooks and arrowheads, whereas shell ornaments are composed of pendants and loops. All the shell tools and ornaments are made of bivalve mollusks and do not occur in large numbers. The finding of these freshwater mollusk remains supports the view that the middle Holocene climate in the Guanzhong Basin may have been warm and moist, which was probably favorable to freshwater mollusks growing and developing in the region.

## Introduction

Mollusks are the second most common members of the animal kingdom and have played particularly important roles in geology since the 1820s, including biological evidence for the hypothesis of continental drift and strong evidence for Lyell to create the term ‘Pleistocene’. Thereafter, most of the fossil mollusk studies have been on the palaeoenvironmental information that can be obtained from studies of mollusk assemblages, rather than on their role in human subsistence [1]–[25]. In fact, mollusk remains are also frequent, often abundant, in late Pleistocene and Holocene archaeological sites [26]. They can supply archaeologists with lots of valuable information, such as food exploitation, trade routes, ornaments and jewellery, tools and containers, etc [27]. Many mollusk species, especially large ones, are still used as foods or tools or decorations. Some species can be used for treatment or prevention of certain diseases and for production of drugs from their shell or soft body [27], [28].

As one of the edible organisms, mollusks provide a considerable amount of food consumed by humans both present and past. Two major classes of them, gastropods and bivalves, are utilized to meet this demand because they are easy to obtain and rich in nutrition [27], [28]. Up to date, there are many studies of the dietary use of freshwater bivalves, terrestrial gastropods and marine mollusks. For example, as summarized by Lubell (2004) [26], Lubell et al. (1976) attempted to test the idea of the contribution of land snails to prehistoric diet in the Holocene Maghreb [29]; Bahn (1983) constructed an interesting argument in favour of Mesolithic snail farming in the Pyrenees [30]; Waselkov (1987) provided global coverage of the pre-1980s literature on mollusks as diet in prehistory [31]; Chenorkian (1989) examined possible dietary contributions of mollusks [32]; and Girod (2003) discussed some of the implications of land snails as food in prehistory [33]. Recently, Gutierrez Zugasti (2011) reported Early Holocene land snail exploitation in Northern Spain [34]. However, most of these studies did not involve freshwater gastropods.

The Chinese Loess Plateau (CLP), located to the northeast of the Tibetan Plateau, is a key region for studies of environmental archaeology, especially for studies of agricultural origins, since archaeological sites particularly of Neolithic age are widely distributed and excavated. Previous studies on prehistoric foods in the CLP focused mainly on large animals and dry farming plants [35]–[39]. Other food resources remain to be investigated.

Mollusks are the most frequent and abundant fossil remains in the loess sequences and Neolithic archaeological sites in the CLP. Most of previous studies on fossil mollusks in the CLP have focused on paleoenvironmental information recorded by mollusk assemblages [5], [11]–[25]. Investigations of fossil mollusks in archaeological sites of the CLP have not been largely reported compared to the paleoenvironmental studies performed on the well-known loess sequences, and how prehistoric humans had used mollusks remains unclear. In this study, we investigate mollusks preserved in middle Neolithic archaeological sites in the Guanzhong Basin (southern CLP) with an objective to examine the exploitation of gastropod and bivalve mollusks, mainly as prehistoric food and furthermore as tools and ornaments, during the middle Neolithic.

## Materials and Methods

**Figure 1:**
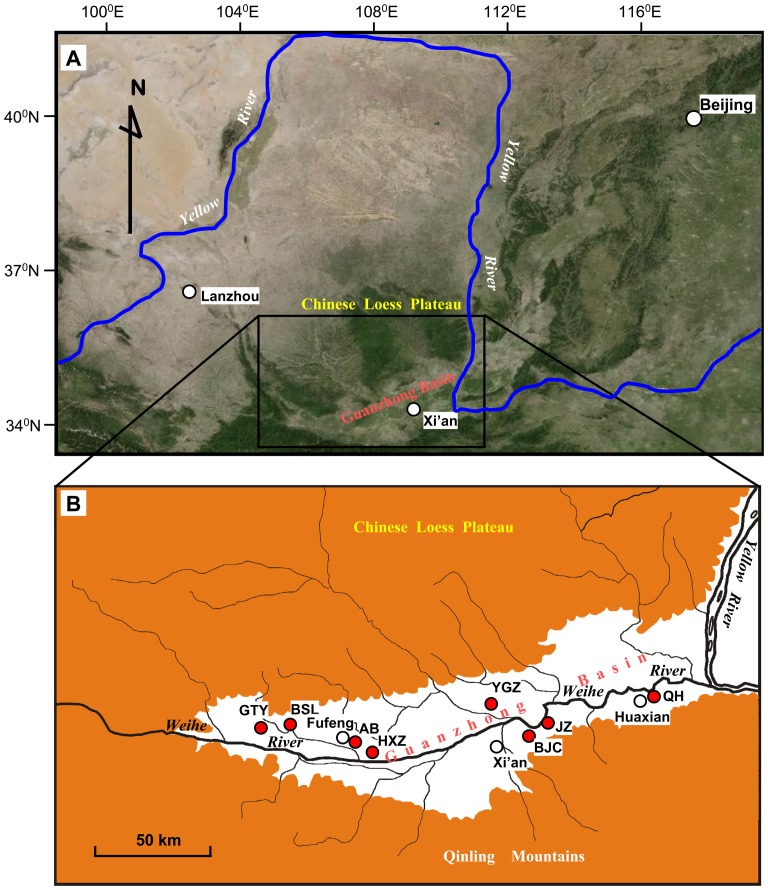
Map showing the studied region and archaeological sites. The white area in panel B is the Guanzhong Basin. GTY–Guantaoyuan; BSL–Beishouling; AB–Anban; HXZ–Huxizhuang; YGZ–Yangguanzhai; BJC–Baijiacun; JZ–Jiangzhai; QH–Quanhu. The map of panel B is modified from Zhang et al. (2010) [39].

The Guanzhong Basin is located in the central part of the Yellow River valley, with the main part of the CLP to the north and the Qinling Mountains to the south (Figure 1). It is crossed by the Weihe River (the largest tributary of the Yellow River) from west to east [38]. It is about 360 km in length from east to west. The altitudes of this region range from about 300 to 600 m above sea level. Although altitudes are significantly lower than the main part of the CLP, this region is also commonly called southern CLP because it is located south of the main part of the CLP and loess deposits are also extensively developed here. The current climate in the Guanzhong Basin is semi-humid and monsoon-controlled temperate zone with mean annual temperature ranging from about 12 to 14°C and mean annual precipitation ranging from about 600 to 750 mm.

The Neolithic sites are extensively distributed in the Guanzhong Basin, making it an important region for studies of prehistoric human activity [38], [39]. As has been summarized by Zhang et al. (2010) [39], Neolithic cultures in the Guanzhong Basin were prosperous and characterized by increasing specialization and complexity in socioeconomic development and can be subdivided into early, middle and late stages [40]–[46]. The Early Neolithic in the Guanzhong Basin is called Laoguantai Culture (ca. 8000–7000 cal. yrs BP). Technology in this culture includes pottery and a variety of stone production tools. The Middle Neolithic is Yangshao Culture (ca. 7000–5000 cal. yrs BP). Prehistoric humans during this period in the region had started to raise domesticated animals and grow crops such as common millet, foxtail millet, possibly hemp (*Cannabis sativa*) and canola (rapeseed, *Brassica rapa*) [47]. The Late Neolithic in the region is synonymous with the Longshan Culture (ca. 5000–4000 cal. yrs BP) in East China.

In the summer of 2007, we undertook investigations of environmental archaeology at six Neolithic archaeological sites in the Guanzhong Basin: Quanhu, Yangguanzhai, Huxizhuang, Anban, Wangjiazui, and Shuigou from east to west (Figure 1). During the investigations, freshwater gastropods and bivalves were found well preserved in pits. As excavations of the Quanhu and Anban archaeological sites finished long ago, we just collected bulk samples from several pits and the surface of a section to keep these sites from being destroyed. All necessary permits for the described field investigations were obtained from Shaanxi Provincial Institute of Archaeology and School of Archaeology and Museology of Northwest University.

**Figure 2:**
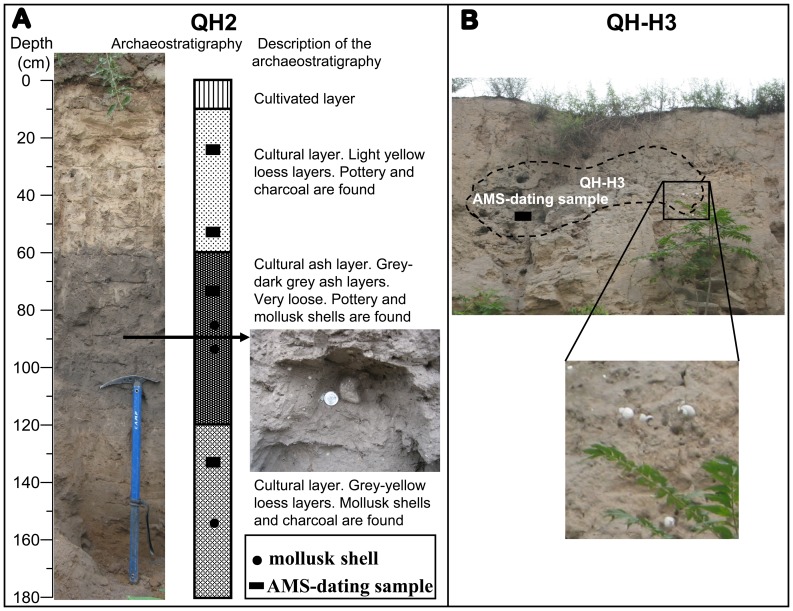
Archaeostratigraphy of the Quanhu (QH2) archaeological site and field photo of the QH-H3 archaeological site. A. Archaeostratigraphy of the QH2 archaeological site, showing from the left to the right stratigraphy photo, archaeostratigraphy with indication of mollusk (solid circles) and age (solid rectangle) sampling layers, description of the archaeostratigraphy of the QH2 site. B. Field photo of the QH-H3 archaeological site with mollusk (solid circles) and age (solid rectangle) sampling layers indicated.

The Quanhu site (N34°31.844′, E109°51.691′), excavated during 1958–1959, is a large prehistoric settlement of about 600 000 m^2^. It is located at Quanhu and Anbao villages (about 8 km east of Huaxian County and 6 km south of the Weihe River) in the second terrace of the Weihe River in the eastern Guanzhong Basin (Figure 1). The archaeological context of this site is from the mid-Yangshao to Longshan Culture (ca. 6000–4000 cal yrs BP) [48]. To keep this site from being destroyed as far as possible, we cleaned a small section (QH2) about 0.30 m wide and 1.8 m high, in which we observed two cultural layers at 0.1–0.6 and 1.2–1.8 m depth and one typical cultural ash layer in between (0.6–1.2 m) (Figure 2). We successively collected bulk samples at 10 cm intervals from the surface of the pit section. A total of 18 samples, each weighing about 200 g, were collected from the QH2 section. Furthermore, bulk samples also weighing about 200 g were collected in two other pits (QH-H1, QH-H3) (Figure 2). In the laboratory, all the bulk samples were washed and sieved using a mesh of 0.5 mm in diameter, and then mollusk shell remains were collected and identified under a set of Leica binocular microscopes. A total of eight radiocarbon samples were collected from the three pits, among which six were from the QH2 profile at depths of 0.2–0.3, 0.5–0.6, 0.7–0.8, 0.9–1.0, 1.1–1.2, and 1.3–1.4 m.

The Anban site (N34°20.749′, E107°54.617′) is located at Anban village, about 4 km southeast of Fufeng County in the western Guanzhong Basin (Figure 1). It is a representative mid-Yangshao to Longshan culture site [49]. A total of one bulk sample weighing about 200 g and one ^14^C dating sample were taken from pit AB-AH2. Further treatment and identification was performed in the laboratory as we did for the samples from the Quanhu site. Mollusk identification was carried out according to descriptions and plates published by Hu et al. (2001, 2007, 2011) and Liu et al. (1979) [28], [35], [36], [50].

**Figure 3:**
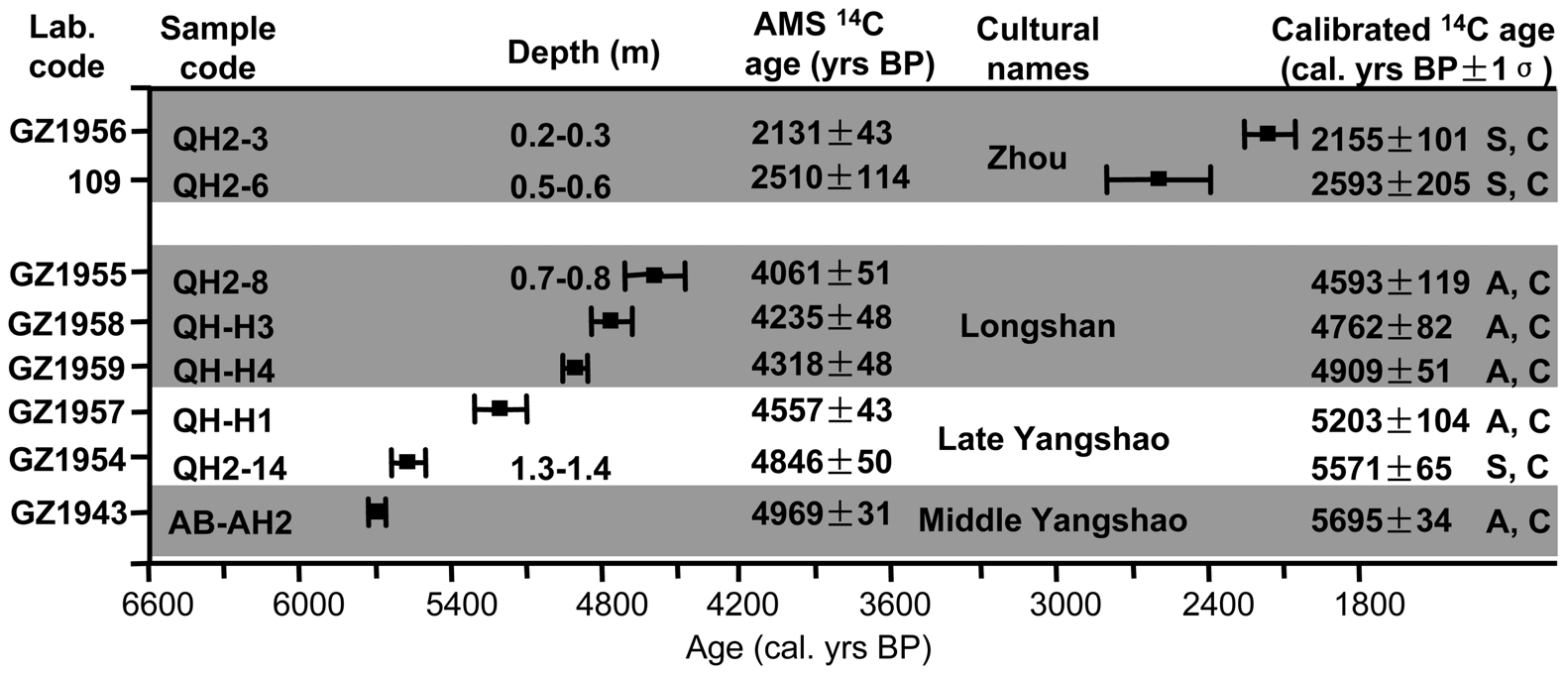
^14^C dates of the Quanhu and Anban archaeological sites in the Guanzhong Basin (modified from Zhang et al., 2010 [39]). The series of GZ-dates are AMS ^14^C dates obtained by the Guangzhou Institute of Geochemistry, CAS and State Key Laboratory of Nuclear Physics and Technology of Peking University. The No-GZ series are conventional ^14^C dates obtained by the Laboratory of Nuclide and ^14^C Chronology of Institute of Geology and Geophysics, CAS. A–ash; S–ancient soil with rich charcoal; C–charcoal.

All the radiocarbon samples from the Quanhu and Anban archaeological sites were measured and have been published by Zhang et al. (2010) [39]. The results related to this study can be seen in Figure 3. The series of GZ-dates in Figure 3 are measured using accelerator mass spectrometry (AMS) in the Guangzhou Institute of Geochemistry, Chinese Academy of Sciences, and the State Key Laboratory of Nuclear Physics and Technology, Peking University. C_AL_P_AL_ was used to calibrate these dates [51], [52]. The No-GZ series in Figure 3 are conventional ^14^C dates obtained in the Laboratory of Nuclide and ^14^C Chronology of the Institute of Geology and Geophysics, Chinese Academy of Sciences. C_ALIB_ R_EV_ 5.0.1 was used to calibrate the dates [53].

In order to further investigate exploitation of mollusk shells by prehistoric humans in this region, we made statistics of types and quantities of shell tools and ornaments in seven archaeological sites, Quanhu, Jiangzhai, Baijiacun, Huxizhuang, Anban, Beishouling, and Guantaoyuan, from east to west in the Guanzhong Basin, based on the excavation reports of these sites [49], [54]–[59].

## Results

**Figure 4:**
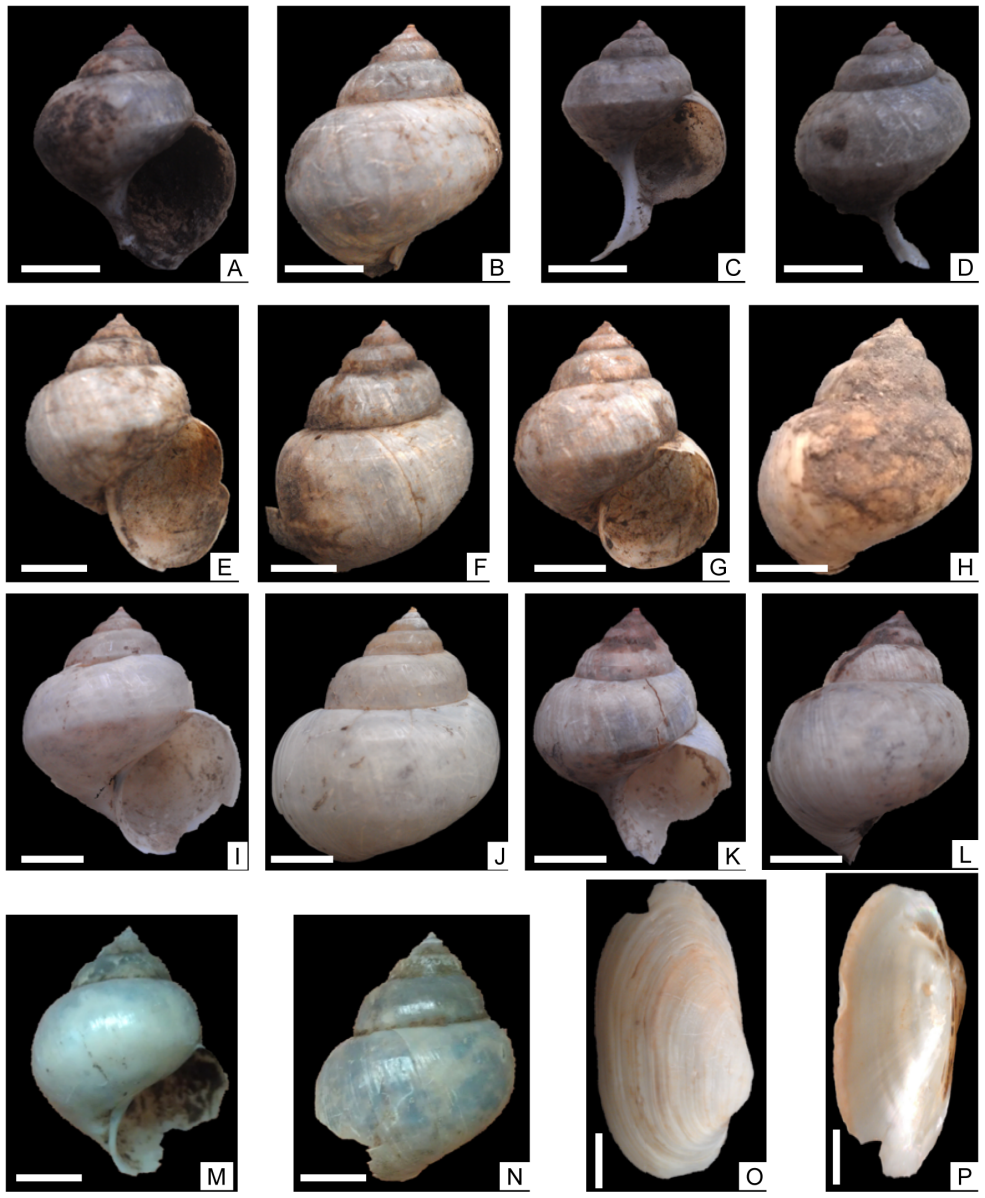
Gastropod and bivalve mollusks found in the Quanhu (QH2 and QH-H3) and Anban (AB-AH2) archaeological sites. A,B, *Cipangopaludina chinensis*, QH2 0.9–0.8 m; C, D, *Cipangopaludina chinensis*, QH2 0.9–0.8 m; E,F, *Cipangopaludina chinensis*, QH2 1.0–0.9 m; G,H, *Cipangopaludina chinensis*, QH2 1.0–0.9 m; I,J, *Cipangopaludina chinensis*, QH-H3; K,L, *Cipangopaludina chinensis*, QH-H3; M,N, *Cipangopaludina chinensis*, QH2 1.6–1.5 m; O,P, *Unio douglasiae*, AB-AH2. Scale bars = 10 mm.

Mollusks are relatively abundant in pits at archaeological sites in the Guanzhong Basin. As recorded in the excavation reports, there were more than 2500 shells in three pits found in the 1958–1959 excavations at Quanhu [58]. Each of the pits investigated in 2007 for this study contained mollusks. All of the shells collected are large in size and well preserved, with whole shells undestroyed and no obvious signs of abrasion (Figure 4).

Pits QH-H1 and QH-H3, each yielded two mollusk shells in 200-g ash samples. One bivalve mollusk was found in the 200-g ash sample from the AB-AH2 pit. Among the 18 samples collected from the QH2 section, only three samples at 1.6–1.5 m, 1.0–0.9 m, and 0.9–0.8 m depths yielded mollusk shells. Each 200-g weighed sample yielded two shells of gastropod and bivalve mollusks. These mollusks were identified as *Cipangopaludina chinensis* (also called *Viviparus chinensis*) and *Unio douglasiae* (Figure 4). Both are freshwater mollusks and have modern representatives. *Cipangopaludina chinensis* is a large gastropod species generally 40 mm in height and 30 mm in width, the largest being 60 mm in height and 40 mm wide [28], [36]. The shell is conical and thin but solid, with a sharp apex and relatively higher spire and distent body whorl. The surface of the shell is smooth with clear growth lines. The aperture is ovoid with a simple outer lip and inner lip. This species prefers freshwater lakes, reservoirs, rivers, paddy fields, and ponds with aquatic grass, creeping at the bottom of the water or on aquatic grasses, as described by Liu et al. (1979) [28]. The optimal water temperature for it to grow and develop is between 20 and 28°C. It will hibernate while water temperature is lower than 10–15 °C or higher than 30 °C. This species is widely distributed in China including the CLP. It is extensively used as diet presently in most places in China because it is delicious and rich in nutrition with high content of protein and low content of fat. Moreover, it is also a medicine used for treatment of digestive disease. It was previously found in the Jiangzhai site of the Guanzhong Basin [57].

*Unio douglasiae* is a bivalve mollusk species of moderate size generally 55 mm in length, 25 mm in height, and 18 mm in width [28], [36]. The shell is relatively thin but solid with a long elliptical shape, generally the length being two times as long as the height. The two sides of the shell are asymmetrical, shorter and rounder in the front as well as longer and relatively narrow and flat in the back. The apex of the shell is large and situated in the front part of the shell. Growth lines are clearly developed and distributed concentrically on the shell surface [28], [36]. As described by Liu et al. (1979) [28], the modern representative of this species is extensively observed in China except on the Tibetan Plateau and the deep interior of the Northwestern China. It is a common mollusk species living in broad water environments including lakes, rivers, reservoirs, and ponds. The soft body of freshwater *Unio* bivalves is widely used as diet due to being rich in nutrition and delicious, and their shells are used as tools and decorations. We found it in the AB-AH2 pit of the Anban site. It has previously been found in the archaeological sites of Guantaoyuan [35], Jiangzhai [57], Yangguanzhai [36], and Quanhu [58] in the Guanzhong Basin, but not in large amounts of individual shells which were used as foods, ornaments and jewellery, and tools. However, in the Gongjiawan site (7500–3500 cal. yrs BP) inside the Qinling Mountains thousands of individual shells were found, indicating main exploitation of mollusks in this place [50].

**Figure 5:**
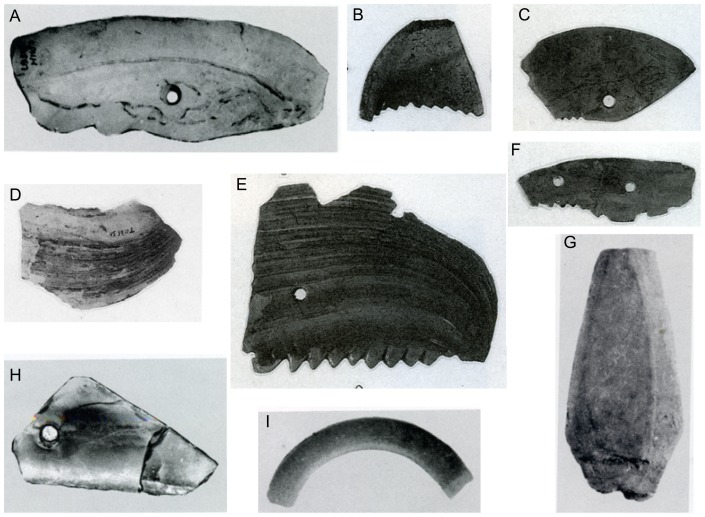
Some shell tools and ornaments from the Quanhu and Baijiacun archaeological sites in Guanzhong Basin (modified after the Institute of Archaeology Chinese Academy of Social Sciences, 1994 [56] and The Archaeology Department of Peking University and the Institute of Archaeology Chinese Academy of Social Sciences, 2003 [**58**]). A,D, shell knife; B,C,E,F, shell reaphook; G, shell arrowhead; H, shell pendant; I, shell loop (part).

**Figure 6:**
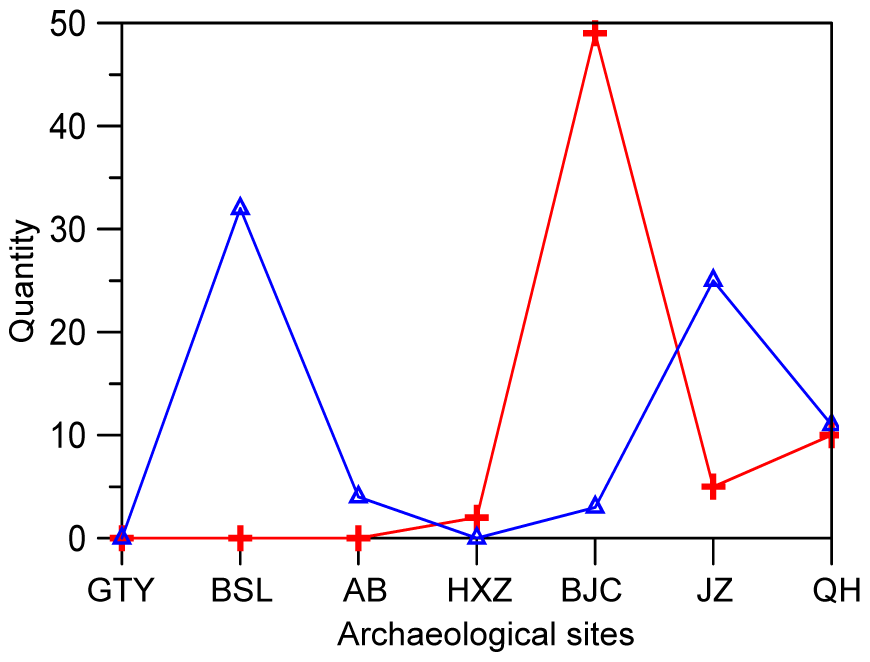
Variations in mollusk shell tool (red curve) and ornament (blue curve) quantities in Guanzhong Basin during mid-Neolithic. The archaeological sites labeled on the X axis are listed according to their longitudes with the westernmost GTY on the left and the easternmost Quanhu on the right. GTY–Guantaoyuan; BSL–Beishouling; AB–Anban; HXZ–Huxizhuang; BJC–Baijiacun; JZ–Jiangzhai; QH–Quanhu.

Mollusk shells used as tools and ornaments were also observed in archaeological sites in the Guanzhong Basin. Shell tools mainly include shell knives and shell reaphooks with the length ranging from 11 to 5 cm and the width from 3.6 to 4 cm (Figure 5). Moreover, several shell arrowheads were found in the Quanhu and Anban sites (Figure 5). Shell ornaments are mainly composed of pendants and a few shell loops (Figure 5). All the shell tools and ornaments are made of bivalve mollusks. Compared with stone and pottery tools and ornaments, shell tools and ornaments are scarce. However, it still can be observed that shell ornaments are generally more common than shell tools in each archaeological site except the middle two sites of Baijiacun and Huxizhuang (Figure 6). Both shell ornaments and shell tools excavated from the eastern sites are much more common than the western sites except Beishouling, with no findings in the westernmost site of Guantaoyuan, which corresponds to warmer and wetter climate conditions in the east than in the west (Figure 6).

Age data determined by AMS and conventional method are given in Figure 3. Results show that the ages are correlated well with the cultural layers and archaeological ages deduced from artefacts. The depth of 1.3–1.4 m in the QH2 profile was given an age of about 5571±65 cal. yrs BP, and the depth of 0.7–0.8 m was given an age of 4593±119 cal. yrs BP. The QH-H1 sample yielded an age of 5203±104 cal. yrs BP and QH-H3 an age of 4762±82 cal. yrs BP. The age of the pit in the Anban site is 5695±34 cal. yrs BP. Thus, all these ages indicate that the mollusks were likely used in the period from about 5600 to 4500 cal. yrs BP, corresponding roughly to the middle Holocene warm period and to the Middle Yangshao to Longshan culture in the Guanzhong Basin.

## Discussion

The study and interpretation of mollusk consumption have been among great concerns and disputes since the first accumulations of mollusks, especially land snails, were discovered in archaeological deposits a century ago [34]. Different interpretations have been proposed to explain the presence of mollusks at archaeological sites. These range mainly from explanations that consider them an anthropic resource [26], [29], [30], [60]–[63] to those supporting the hypothesis of natural accumulations [64], [65], as summarized by Gutierrez Zugasti (2011) [34].

Here we attribute the mollusks found in the archaeological sites of the Guanzhong Basin to prehistoric food remains, thus supporting an anthropic origin. Evidence supporting this proposition is as follows: 1) all of the shells collected are well preserved in pits with whole shells undestroyed and no obvious signs of abrasion; 2) all the mollusks were found in pits where no water-reworked deposits were observed, but all the mollusks are freshwater gastropods and bivalves, large in size, indicating that they are not in situ fossils deposited in the pits; 3) all the mollusks have modern representatives, which can be observed in rivers, reservoirs and paddy fields in the studied region and they are still delicious foods nowadays; 4) As described in the excavation reports of the Quanhu and Anban archaeological sites [49], [58], mollusk shells are frequently recovered in direct association with many mammal bones, lithic artifacts, and pottery, which are indications of human activities. Based on these observations, we suppose that they were used as foods by prehistoric populations who discarded them into the pits after the flesh was consumed, supporting those explanations that mollusks in archaeological deposits are attributable to an anthropic resource [26], [29], [30], [60]–[63].

Since land snails are probably one of the first domesticated animals, as suggested by Fernández-Armesto (2002) [66] and argued by Lubell that this is almost impossible to test [26], [67], increasing attention has been paid to their importance in archaeology. Mollusks as remains of prehistoric meals in the late Pleistocene and Holocene sites throughout the Mediterranean region were among the well-studied examples, as summarized by Lubell (2004) [26]. In China, mollusks as prehistoric food have been reported at least as early as 12000 years ago from the Yuchanyan site in Southern China [68]. However, extensive use of mollusks as food may start from the Holocene as seen in a great deal of Neolithic archaeological sites [49], [54]–[59]. In the Guanzhong Basin there are numerous Neolithic archaeological sites containing mollusks, such as those investigated in this study. Archaeological excavations showed possibilities of mollusks as food, tools and ornaments by prehistoric humans in the Guanzhong Basin. Occurrence of freshwater mollusks in the studied sites indicates that there should have been freshwater bodies such as rivers, reservoirs or paddy fields near the studied region during the warm and humid middle Holocene, providing a suitable niche for them to grow and develop. This can be supported by the discovery of rice in the Quanhu archaeological site in the Guanzhong Basin [39]. The Quanhu site is near the floodplains of the Weihe River [58], so it is convenient for the prehistoric humans to have used water from the Weihe River to cultivate rice in the middle Holocene when global climate was warmer and more humid.

It has been pointed out by several researchers that mollusks should be considered a secondary and non-staple resource because they are not available all year [29], [30]. However, recent study proposes that mollusks can be a very productive resource, providing plentiful calories using only a small amount of energy in gathering and preparing them [63]. For this reason, it may be considered that at certain times of the year, they could have played an important role in the nutrition and subsistence of human groups. However, in the archaeological sites investigated in the Guanzhong Basin, all the mollusk shells were not in very large quantity, indicating that mollusks were most likely an occasional collection and consumption in the investigated places in the Guanzhong Basin and never as staple food. In fact, *Cipangopaludina chinensis* should really be considered as a seasonal food resource since its activity mainly focuses on seasons during which water temperatures are higher than 10–15 °C and lower than 30 °C, Therefore, the most possible collection season should be concentrated on months from April to October in the Guanzhong Basin. Based on these observations and the information currently available, we deduce that mollusks in the archaeological sites of the Guanzhong Basin appear to represent occasional gathering and consumption events.

Previous studies had shown that the prehistoric populations had exploited rice, millet, wheat, barley, oats, soybean, and buckwheat as food in the Guanzhong Basin during the Mid-Neolithic [37], [39], and even invented noodles in Northwestern China 4000 years ago [69], indicating that food diversity has appeared in the western CLP and Northwestern China during the Mid-Neolithic [37]. Our study indicated that they also started to eat mollusks during the Mid-Neolithic. Mollusks are sluggish or immobile and thus are relatively easy to collect. Moreover, mollusks especially freshwater ones are rich in nutrition and edible. Thus, mollusks are potentially good foods in that they have high energetic returns and low cost in capture and handling. Indeed, mollusks, compared with large animals, have long been of importance to human societies not only being used as food, but also as medicine, tools, personal ornamentation, currency in trade, and etc. Two major classes of them, bivalves and gastropods, are utilized to meet this demand. Our study shows that freshwater bivalve and gastropod have been exploited as food, probably not staple food, by the prehistoric populations in the Guanzhong Basin, Northwestern China, during the middle Holocene. Thus, our study combined with previous studies show the occurrence of food diversity in North-Central China during the Mid-Neolithic. However, the reason for the occurrence of food diversity remains to be studied.

Exploitation of mollusks by prehistoric human groups is not limited to foods. The appearance of ornaments such as pendants since the Paleolithic marks an important step in the evolution of human behavior. Comparatively standard ornament forms were made of shell, tooth, ivory, or stone [70]. Mollusk shells as ornaments have been found in the Neolithic archaeological sites in Israel [71]–[73]. In China, mollusk shell ornaments and tools were also found in the Mid-Neolithic archaeological sites in the Guanzhong Basin [49], [54]–[59]. For example, shell knives were discovered from the Quanhu, Jiangzhai, Baijiacun, and Huxizhuang archaeological sites (Figure 6), which were used by prehistoric humans as agricultural tools. One of them was incompletely preserved with 6.2 cm long and 4.2 cm wide [58]. As an ornament, shell pendants were found in the Quanhu, Jiangzhai, Baijiacun, Anban, and Beishouling sites (Figure 6), which was made of thick freshwater mollusk shells, 52 mm long. Moreover, shell loops and shell arrowheads (perhaps used for hunting) were also excavated in the Quanhu and Anban sites in the Guanzhong Basin [49], [58]. All the shell tools and ornaments are made of freshwater bivalve mollusks, no marine ones, indicating that they were likely obtained from adjacent water bodies. Although the shell tools and ornaments are not large in amount either, their occurrence in the investigated archaeological sites indicated that the humans in the Guanzhong Basin had relatively widely exploited mollusk shells, not only as food but also as tools and ornaments. As shown in Figure 6, shell tools and ornaments from the east sites in the Guanzhong Basin are more in Quantity than from the west sites except the Beishouling site, indicating that the middle Holocene climate in the Guanzhong Basin may be warm and moist characterized probably by warmer and wetter climate conditions in the east than in the west [38], favorable to mollusks growing and developing in the Guanzhong Basin. In fact, the warm and moist climate conditions during the Mid-Neolithic have been revealed by previous studies. A Holocene mollusk record from the CLP showed that thermo-humidiphilous species, *Macrochlamys angigyra* and *Punctum orphana*, were dominant during the middle Holocene, showing warm and moist climate condition prevailed [20]. The pollen records from both the western and eastern Guanzhong Basin of the middle Weihe valley in Northwestern China show that the vegetation reverted to sparse-wood grassland dominated by arboreal plants during this period [74]. All of these evidence show clearly warm and humid climate conditions dominated during the middle Holocene.

It should be pointed out that the periods of 5600–5000 yrs and 5000–4500 yrs correspond to Late Yangshao Culture and Early Longshan Culture, respectively, which may have different shell tools or ornaments. However, we can not discuss the evolution of the shell-tool shapes and their relationship with environmental/climatic changes based on the material presented in this study because the present sample resolution and materials are not enough to address this issue. It remains to be studied by high resolution data and mollusk shells.

## Conclusion

Mollusks are abundant remains in the archaeological sites in the Guanzhong Basin. We report exploitations of mollusks by prehistoric populations in this region in Mid-Late Neolithic age, mainly as subsidiary food and furthermore as tools and ornaments. Results showed that freshwater gastropods and bivalves, *Cipangopaludina chinensis* and *Unio douglasiae*, are the main species excavated from the archaeological sites in the Guanzhong Basin. They were deposited in pits which had not been reworked by water, and are well preserved with no signs of abrasion, indicating that they are not in situ fossils deposited in the pits. Their modern representatives can be observed in rivers, reservoirs and paddy fields in the studied region and used as food presently. Moreover, mollusk shells are frequently recovered in association with mammal bones, lithic artifacts, or pottery. These lines of evidence indicate that the mollusks found in the archaeological sites in the Guanzhong Basin are the remains of prehistoric meals. However, these mollusk remains may not be staple food in the Guanzhong Basin since they are not in large quantities. Shell tools include many shell knives, shell reaphooks and several shell arrowheads, whereas shell ornaments are mainly composed of pendants and furthermore a few shell loops.

It should be pointed out that there are plenty of Neolithic archaeological sites in China, which contain abundant mollusks remaining to be investigated and studied. We only investigated mollusk remains in the archaeological sites in the Guanzhong Basin. More studies are needed to focus on the mollusks in other archaeological sites, which could provide new evidence and knowledge for our understanding of mollusks and prehistoric humans. Forthcoming studies need to address other questions related to the role of mollusks in the subsistence strategies of human groups, such as the collection patterns, the forms of preparation and consumption.
